# Body Composition in Adolescent PKU Patients: Beyond Fat Mass

**DOI:** 10.3390/children9091353

**Published:** 2022-09-04

**Authors:** Albina Tummolo, Rosa Carella, Giulia Paterno, Nicola Bartolomeo, Massimo Giotta, Annamaria Dicintio, Donatella De Giovanni, Rita Fischetto

**Affiliations:** 1 Department of Metabolic Diseases and Clinical Genetics, Giovanni XXIII Children Hospital, Azienda Ospedaliero-Universitaria Consorziale, 70126 Bari, Italy; 2 Interdisciplinary Department of Medicine, University of Bari Aldo Moro, 70124 Bari, Italy

**Keywords:** PKU, adolescence, bioelectrical impedance analysis, muscle mass, branched-chain amino acids, quantitative ultrasound

## Abstract

There is a lack of evidence on the impact on body composition of high protein intake and types of protein substitutes in PKU patients—particularly in adolescents, who are more inclined to dietary transgressions. In this observational, cross-sectional study, PKU patients were observed during prepubertal age (p) or after the pubertal spurt (P), assessing body composition and bone quality and correlating these parameters with dietary compliance and types of protein substitutes. Anthropometric and dietary data were evaluated together with bioelectrical impedance analysis (BIA), quantitative ultrasound (QUS) and branched-chain amino acids (BCAAs). A total of 36 patients (16 males, 17 prepubertal and 19 post-pubertal; mean ± SD age 11.4 ± 3.9 years) were included. A higher BMI was observed in adolescents (*p*-value: 0.018). The BIA revealed a significant increase in total body water (TBW) and muscle mass (MM) in P subjects either compliant (*p*-value: 0.001) or non-compliant with the diet (*p*-value: 0.001). MM content correlated with increased Phe intake (r = 0.63; *p* < 0.001). In the subgroup of five patients taking L-AAs and glycomacropeptides (GMPs), BCAA values tended to be lower than those taking only L-AA mixtures, with a significant trend for valine. Maintenance of body composition parameters within the normal range—for both fat and muscle mass—and levels of BCAAs can be helpful in reducing the risk of becoming overweight in adulthood. Further studies are needed to confirm these findings.

## 1. Introduction

Phenylketonuria (PKU) is a rare autosomal recessive inborn error of phenylalanine (Phe) metabolism caused by the deficient activity of Phe hydroxylase (PAH), which is needed to convert Phe into tyrosine. When left untreated, patients with PKU develop severe and irreversible neurological damage, growth deficiency, eczema and skin and hair hypopigmentation [1].

The mainstay of treatment involves a Phe-restricted diet, composed of strict control of natural protein intake, administration of protein substitutes (PE) derived from Phe-free amino acids (L-AAs), large neutral amino acids (LNAA) or low-Phe glycomacropeptide (GMP) [2,3] and the use of special low-protein foods. The primary aim is to maintain blood Phe levels within a therapeutic target range in order to prevent neurological sequelae [1] while ensuring nutritional requirements are met for normal growth development [2,4,5]. To obtain these endpoints, the daily protein intake of individuals with PKU exceeds the WHO/FAO/UNU protein intakes of the general population [6].

L-AAs are special formulas containing synthetic mixtures of amino acids devoid of Phe and supplemented with tyrosine—the amino acid that is lacking in PKU. A variety of commercial products are available to meet patients’ preferences for taste and palatability [3].

GMP is a natural Phe-free protein source derived from whey that contains small amounts of Phe. However—given the low Phe content—GMP has been used for PKU patients, and many studies have identified no adverse events and have demonstrated its efficacy in maintaining serum levels of Phe—mainly thanks to the palatability of the formulations [3,7].

The impact of a restricted diet regimen on both short- and long-term growth [8] requires ongoing evaluation [9,10]. Although Body Mass Index (BMI) is widely used as the outcome measure of growth, it has clear limitations. The relative contribution of lean and fat mass to body weight cannot be assessed. Therefore, based on BMI, some patients may appear to be relatively weight-stable, despite actually changing in terms of their underlying fat/lean ratio.

Bioelectrical Impedance Analysis (BIA) offers a rapid, simple, noninvasive and cheap way of assessing body composition in children and adolescents [11].

Adolescence, in particular, is a period of growth and maturation, characterized by changes in body composition—mainly in the location and amount of body fat and changes in anabolic effects, with reduced insulin sensitivity [12,13]. In PKU patients, many studies [8,14,15] have emphasized the risk of higher fat mass percentages related to diet content and lifestyle—being particularly evident in females. PKU adolescents may be particularly exposed to body composition deviations because of their diet [16,17,18] and their higher frequency of diet transgressions [7,19,20]. Some studies have found that PKU subjects with poorer metabolic control due to low compliance to diets are more likely to be overweight than others, but the relative contribution of fat and muscle to their being overweight has not been assessed [16,21,22].

The role on muscle body mass of a high protein diet in PKU patients has been poorly investigated, as well as the effects of the different protein substitutes used to meet their daily protein requirements on their body composition and bone mineralization.

In our study, we aimed at assessing body composition and bone quality in adolescent PKU patients, comparing them with prepubertal PKU patients. We also correlated body composition and bone quality parameters with compliance to diet and type of protein substitutes: L-AAs with or without GMPs.

## 2. Materials and Methods

### 2.1. Patients

We performed an observational, cross-sectional study on a cohort of patients affected by PKU. The inclusion criteria were diagnosis by neonatal screening and confirmation by molecular analysis. The study was conducted between May 2017 and January 2022. The exclusion criteria were concomitant chronic disorders altering bone mineralization and/or nutritional conditions and/or timing of puberty (precocious or delayed puberty were excluded). The type of PKU was assigned based on the PKU European Guidelines [1]. Written informed consent was obtained from parents. All procedures were in agreement with the guidelines of the Declaration of Helsinki on Human Experimentation.

Patients were assessed either during prepubertal age (prepuberal group hereafter referred as group “p”) or after the pubertal spurt (pubertal group referred as group “P”). The Tanner stage (prepubertal, Tanner 1; pubertal, Tanner 2 to 5) was used to determine pubertal progression by physical examination [23].

A subgroup of 5 patients was observed during both periods.

Anthropometric parameters were assessed with the same balance and altimeter weighting, measuring patients in light clothing. BMI was calculated as the weight/height^2^ ratio; normal values of the BMI Z-score range between +1.0 and −1.0, according to the World Health Organization (WHO) charts [24].

All patients were treated with a diet regimen restricted in Phe and supplemented with L-AAs, and in five cases, even took GMP supplementation. A trained metabolic dietitian analyzed the dietary data from each patient using Winfood Pro software (version 3.0.0, 2011, Medimatica Srl, Teramo, Italy). Dietary protein intake, expressed in grams per kilograms per day, was extrapolated from FAO/WHO/UNU recommended safe levels [6], with an extra percentage of total protein intake necessary to support growth, differing by age [25,26]. Energy intake was expressed as kcal/Kg/day and, for PKU subjects, was comparable with the estimated average requirements of the general population [27]. Compliance to the diet was evaluated through the mean Phe levels resulting from the weekly dried blood spot (DBS) tests performed over the previous 6 months; values were directly retrieved from the laboratory database. Target levels were: 120–360 µmol/l for patients aged <12 years and 120–600 µmol/l for those older than 12 years of age, according to the European Guidelines [1]. Dietary compliance was defined as poor if mean DBS Phe levels were higher than the target level or good if mean dried blood spot Phe levels fell within the target Phe level. All our patients took the entire prescribed quantity of synthetic proteins even if they were poorly compliant to the diet. The daily amount of GMP was calculated as 70% of the total protein equivalents (PE), expressed in grams of the product used [28]. Additionally, the percentage of GMPs—expressed as g/kg/day—over total PE intake (g/kg/day) was calculated. Serum samples were assayed for branched-chain amino acids (BCAA) by liquid chromatography (LC)–MS/MS [29].

### 2.2. Body Composition

BIA measurements were performed by a trained study assistant. A multi-frequency (20 kHz and 100 kHz) BIA device using an eight-point tactile electrode system (Inbody 230, Biospace Corp., Seoul, Korea) referred to as BIA8MF was used. The performance characteristics of the instrument and the procedures used have been already described [30]. Body composition parameters, calculated by the manufacturer’s software (Lookin’Body 120, Biospace Corp., Seoul, Korea) were: fat mass (FM; Kg), percentage of body fat (PBF), total body water (TBW; Kg), waist–hip ratio (WHR) and muscle mass (MM; Kg). The machine provided normal ranges of BIA parameters for the general population according to gender and age.

### 2.3. QUS (Quantitative Ultrasound Scan)

A calcaneus QUS SONOST 3000 (Osteosys, Korea) was used to calculate the bone quality index (BQI) and the BQI SDS. The BQI was derived from the broadband ultrasound attenuation (BUA, measured in dB/MHz) and the speed of sound (SOS, measured in m/s). The BUA reflects bone structure and is directly related to temperature; the SOS reflects bone mineral density and is inversely related to temperature. Correlation coefficients (α, β) combined with BUA and SOS allow one to obtain the BQI (BQI = α × SOS + β × BUA) [31].

### 2.4. Statistical Analysis

Thirty-six patients were recruited during the observation period (17 prepubertal and 19 pubertal).

For a two-sample pooled *t* test of a normal mean difference with a two-sided significance level of 0.05, assuming a common standard deviation of 0.3 g/kg, group sample sizes of 17 and 19 have a power of 0.82 to detect a mean difference of 0.3 g/kg in total protein intake [30] between the Prepubertal and Pubertal group.

Continuous variables were expressed as the mean and standard deviation (SD) for normally distributed parameters or the median and interquartile range (IQR) in the case of skewed data distribution. Shapiro–Wilk statistics were used to test normality. Differences in continuous variables between groups were compared using a Student’s *t*-test for normally distributed parameters or a nonparametric Mann–Whitney U Test otherwise.

Categorical data were expressed as frequency and percentage, and the Chi-square test or Fisher’s exact test was used to compare the two groups.

Multivariable generalized linear models were applied to evaluate the effects of puberty and compliance to diet—adjusted for sex—on the BIA and QUS parameters and on levels of BCAA. The results are shown as least square means with their standard errors; pairwise multiple comparisons were adjusted according to the Tukey correction.

All tests of statistical significance were two-tailed, and *p*-values less than 0.05 were considered statistically significant. Statistical analysis was performed using SAS/STAT^®^ Statistics, Version 9.4 (2013), SAS Institute Inc., Cary, NC, USA.

## 3. Results

### 3.1. Comparison of BIA and QUS Parameters in Different Subgroups

A total of 36 patients (17 p and 19 P, mean ± SD age 11.4 ± 3.9 years) were included in the study. Of them, five were observed over both periods, for a total of 41 observations. The demographic and dietary characteristics of the entire group and of the two subgroups are shown in Table 1. Nine out of thirty-six patients (25%) were considered non-compliant with the diet, based on the mean of the Phe values of the previous six months: 18% in the p group and 32% in the P group (*p*-value: 0.451).

Regardless of dietary compliance, considering the whole group of patients, a significant increase in BMI was observed in the P group compared to p (18.06 ± 2.8 vs. 22.47 ± 3.78 (*p*-value: <0.001)). However, in adolescents, the PBF was significantly reduced (28.46 ± 14.62 vs. 19.17 ± 12.93, *p*-value: 0.049), whereas the BQI was significantly increased (53.73 ± 7.27 vs. 71.5 ± 17.49, *p*-value: 0.033; data not shown).

We performed a comparison of BIA and QUS parameters between males and females, regardless of their age (Table S1); no statistically significant difference was found in any parameters. Considering dietary compliance, a multivariate analysis adjusted for sex showed a significant increase in BMI between the prepubertal and post-pubertal group (*p*-value: 0.018; Table 2, a vs. c). No statistically significant differences in the amount and percentage of fat mass was observed in any of the subgroups analyzed (Table 2). The same was observed for the BQI and SDS-BQI. Within the prepubertal group, being compliant or non-compliant to the diet did not produce statistically significant differences in the QUS and BIA parameters (Table 2, a vs. b). On the other hand, in the P group, following or not following the diet resulted in a statistically significant difference in TBW (*p*-value: 0.001) and MM (*p*-value: 0.001; Table 2, c vs. d). These values are also higher than the upper limit of the mean normal values for prepubertal and post-pubertal children (Table 2).

Concerning those compliant to the diet, the comparison between the two groups showed an increase in TBW (*p*-value: <0.001), WHR (*p*-value: 0.01) and MM (*p*-value: <0.001) in the P group (Table 2, a vs. c).

Similarly, among those non-compliant to the diet, TBW and MM were significantly lower in the p than in the P group (*p*-value: <0.001), highlighting the change in the amount of water and muscle tissue after puberty, regardless to the adherence to diet (Table 2, b vs. d). No difference among the subgroups for BCAA values were found. For all parameters, the “sex” adjustment factor was not significant.

### 3.2. Comparison of BIA and QUS Parameters before and after the Pubertal Spurt

An analysis of five patients before and after the pubertal spurt showed an increase in BMI in all cases. Within the BIA parameters, FM remained almost stable in Patient 1 and Patient 4, while an increase in FM occurred in the others. The PBF presented a variable trend. The parameters that increased in all cases were TBW and MM, which were found to always be higher after puberty. The WHR was also higher in all patients in the post-pubertal period. For QUS parameters, SDS-BQI remained almost stable in two cases (Patients 4 and 5), but decreased in the remaining three patients (Table 3).

### 3.3. Comparison of BIA and QUS Parameters According to Type of Protein Substitute

**Figure 1:**
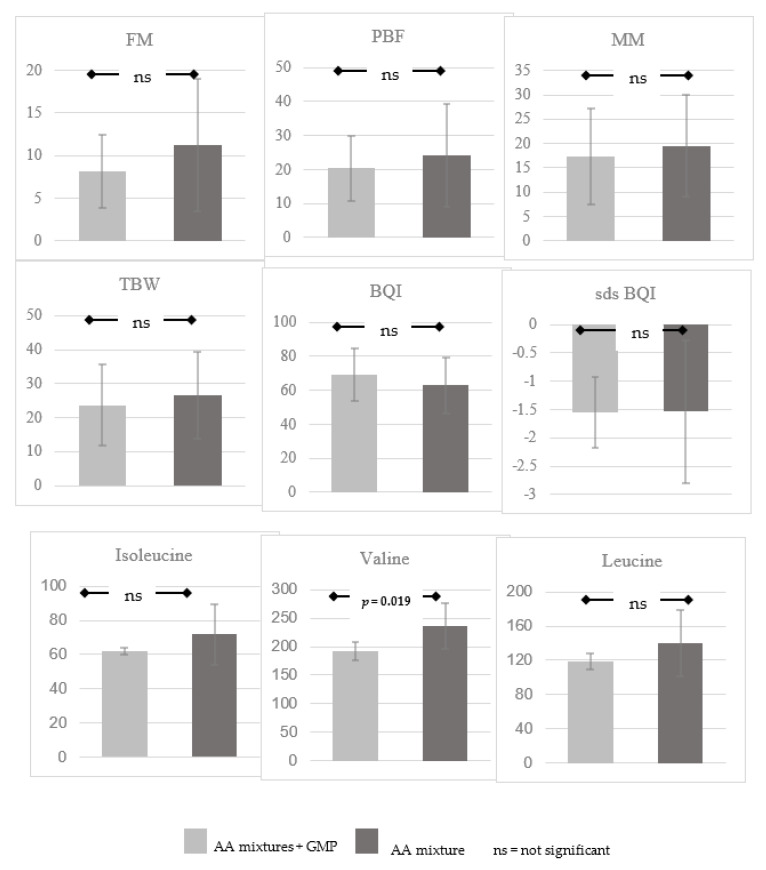
Comparison of parameters according to type of protein substitute.

An evaluation of BIA and QUS parameters and BCAA levels in relation to the type of synthetic protein consumed—L-AAs alone (31 patients) or with GMP (five patients)—showed no significant differences in BIA and QUS parameters, although a trend toward a higher BQI and a lower FM and MM was observed in the second subgroup. The group of patients taking GMP with L-AAs tended to also have lower branched-chain amino acid values, with statistical significance for valine (*p*-value: 0.01; Figure 1).

### 3.4. Correlation of BIA and QUS Parameters with Protein Intake and Compliance to Diet

**Figure 2:**
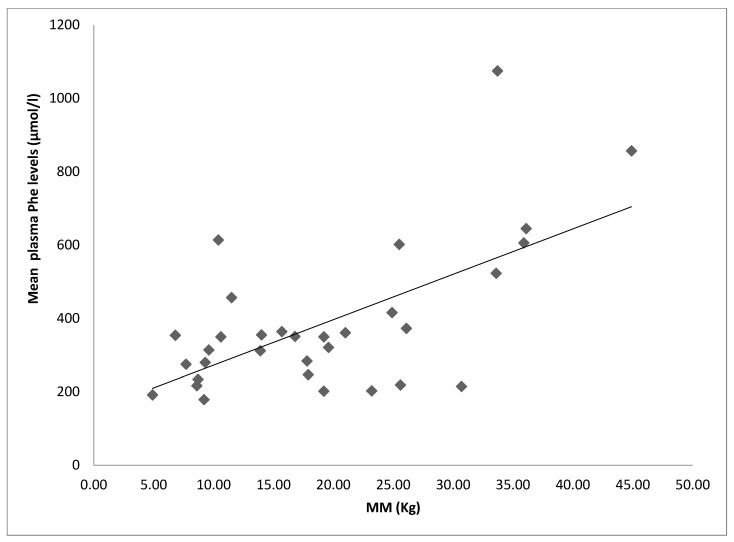
Correlation between mean plasma Phe levels and muscle mass (MM). r = 0.63 *p* < 0.001; Analysis only possible for 32 out of 36 patients because of missing BIA values.

The only significant correlations (*p*-value < 0.05) identified were: TBW and MM with dietary Phe intake (respectively r: 0.47; 0.46) and WHR with increasing mean Phe values (r: 0.43). No significant correlations with the other BIA parameters or with the BQI were retrieved (data not shown). The increase in MM correlated significantly with average Phe values (Figure 2).

## 4. Discussion

Our study showed a higher BMI in post-pubertal than prepubertal PKU patients, due to significant increases in TBW and MM in both compliant and non-compliant to diet subjects—in this last case, also exceeding to the normal range for the general population. Muscle mass content correlated with increased plasma Phe levels and therefore with diet compliance.

In the subgroup of patients taking L-AAs and GMPs, we found no statistically significant differences in BIA and QUS values compared to the group taking only L-AA mixtures; however, the branched-chain amino acid values tended to be lower in the GMP group than in those who took only L-AA mixtures, with a significant trend for valine.

Body composition changes in the human body with age and is also gender-specific [32]. This is secondary to reproductive and endocrine changes in the body [33]. During puberty, hormonal fluctuations as well as a rapid growth in body size are accompanied by marked changes in body composition [34]. This situation often correlates with the development of excessive weight and obesity in children [35], although even the MM increases after puberty [36]—but this is generally not considered a harbinger of later-life metabolic disturbances.

In our cohort of patients, we did not find an increase in FM after puberty—in contrast with what has been reported by other studies [13,14,15], with no differences between males and females.

It can be hypothesized that the influence of diet on the body composition of these subjects overtakes the effects of sexual hormones that are responsible for the different changes in body composition between the two sexes after puberty [37,38,39,40].

In our sample, the increased BMI in post-pubertal patients was sustained by a higher muscle mass and improved bone quality index. In this context, BIA provided a more accurate assessment than BMI on the muscle/fat ratio.

This increase is explained by studies reporting greater protein retention at this age. After puberty, selective stimulation of whole-body protein synthesis by GH, IGF-1 and androgenic hormones may explain the higher protein gain during this period [41].

Significant evidence from large, high-quality randomized control trials demonstrates that the higher protein content of formula milk, more than breast milk, is associated with adverse infant and child outcomes [42,43]. The role of protein intake on adiposity beyond the weaning period has also been investigated [37,38,39]; Gunther [44] and Assmann [45] reported that higher protein intake exceeding physiologic requirements, particularly between the age of 9 and 15 years, is associated with higher fat-free mass (FFM). The growth hormone/insulin-like factor 1 axis may be stimulated by excessive protein intake and drive the early differentiation and proliferation of adipocytes [46].

PKU patients can be considered a vulnerable group for the above metabolic abnormalities. In fact, to ensure normal growth, PKU children are prescribed a total protein intake that exceeds the WHO/FAO/UNU 2007 safe levels of proteins [1].

Special attention should be paid to older children and adolescents, who have autonomy in dietary choices and are often inclined to dietary transgressions, which in case of normal protein substitute intake, is associated with an even higher daily protein intake.

GH, IGF-1 and sex steroids all markedly increase during puberty and their actions are amplified mutually as they control not only increases in muscle mass but also affect the mineralization of the skeleton. The synergistic actions of these anabolic hormones appear to be most significant during the finite years of puberty [47,48].

An increase in bone density during the adolescent period has also been reported, with both genders reaching peak bone gain after puberty [49]. This higher protein retention may explain the increasing bone quality observed by us in the present and in a previous study by our group [31], which has also been confirmed by other authors [50].

In classical PKU, the protein substitutes L-AAs and/or GMP provide up to 80% of dietary protein requirements and are essential to ensure metabolic stability and growth. Protein substitutes, while meeting the protein requirements for cellular function and growth, have several pharmacological and physiological functions [51,52]. The amino acid profile is different for each protein substitute, with variations in amino acid patterns as well as in the amount of essential and non-essential amino acids per 100 g of PE. Additionally, their relative absorption rates and bioavailability are not fully understood [53].

In currently available GMP formulas, the amount of Leucine on average exceeds that of the most common L-AAs formulas (196 vs. 121 mg/g PE), while the amount of Isoleucine is comparable (71 vs. 77 mg/g PE) and there is a lower amount of Valine (60 vs. 89 mg/g PE). Modifications in GMP composition, however, do not determine statistically significant differences in BCAA plasma levels compared to the average values of BCAAs in patients taking L-AAs—except for a decrease in Val levels [54], as confirmed by our study. However, this seems to be a beneficial effect of GMPs, since recent studies associate high levels of Valine with increased oxidative stress and the onset of Type 2 Diabetes [55].

In this context, other studies have reported that elevated levels of plasma BCAAs may be associated with obesity [56] and insulin resistance [57,58,59]. However, the relationship between BCAA and detailed parameters of body composition has yet to be reported.

Acute increases in plasma amino acids, linked to the absorption profile of L-AAs, worsen insulin sensitivity. L-AAs are unable to replicate the physiological actions of whole proteins, being directly absorbed from the small intestine [60]. Substantial evidence suggests that the ingestion of large doses of L-AAs increases amino acid oxidation and nitrogen excretion, decreasing their availability for cellular functioning—whereas GMP lowers the rate of amino acid absorption and improves nitrogen retention [61].

Ney et al. [62] and van Calcar et al. [61] suggested that GMP may induce a slower and more sustained release of amino acids, leading to a more physiological availability of amino acids, leading to improved growth and body composition.

There have been few studies investigating changes in body composition in PKU patients taking L-AA and/or GMP. A 3-year prospective study by Daly et al. [21] compared the effect of GMP vs. L-AAs on body composition and growth in 48 PKU children between 5 and 16 years of age. Results showed that children in the GMP group had improved lean body mass (LBM), with decreased fat mass and % body fat—possibly secondary to the high content of BCAA in GMP. A higher LBM content may be supposed; however, this study is not based on the new GMP compositions and does not report information about MM content and the measurement of plasma BCAA.

In another study, Huemer et al. [63] measured growth and body composition over 12 months in 34 children with classical PKU. Total protein intake was 124% of the German recommended daily allowance. A significant correlation was found between LBM and natural protein intake, in keeping with our results—even though the possible implications of a particularly high protein intake in these patients were not discussed.

Limitations of our study include the low number of patients in the GMP group compared with those also administered with L-AAs and the lack of insulin dose as a marker to correlate with BCAA levels. On the other hand, this study has some strengths: The inclusion and exclusion criteria for patient selection and the inclusion of independent variables (covariates) in a multiple regression model [64] reduced the selection bias problems typical of cross-sectional observational studies. This is the first study highlighting the importance of assessing muscle mass content, beyond fat mass content, in helping assessing nutritional risks—particularly during adolescence and under non-compliance conditions. Furthermore, this represents the first evidence for a potential role of GMPs in keeping plasma BCAA levels lower, thanks to their more physiological amino acid absorption profile.

## 5. Conclusions

In conclusion, BIA, performed regularly, provides more complete elements than BMI for the evaluation of the nutritional status of PKU patients—especially in adolescence. In this period, MM increased compared to FM—especially in subjects with a low compliance to the diet. Maintenance within the normal range of parameters of body composition, for both fat and muscle mass, and levels of branched-chain amino acids can be helpful to reduce the risk of becoming overweight in adulthood. Further studies involving a greater number of patients and a correlation between total protein intake, use of different protein substitutes in PKU diets and the risk of metabolic syndrome in adult life are needed to support these results.

## Figures and Tables

**Table 1:** Demographic and dietary characteristics of the patients.

	Total (n = 36)	p Group (n = 17)	P Group (n = 19)	*p*-Value
Males (%)	16 (44%)	6 (35%)	10 (53%)	0.296
Age (years)	11.4 ± 3.9	8.1 ± 2.2	14.4 ± 2.4	**<0.001**
Phe restriction + LAA mixtures + GMP	5 (14%)	3 (18%)	2 (11%)	0.650
Total protein intake (g/Kg/day)	1.24 ± 0.30	1.39 ± 0.31	1.10 ± 0.21	**0.002**
Natural protein intake (g/kg/day)	0.39 [0.22–0.75]	0.41 [0.29–0.79]	0.32 [0.19–0.70]	0.288
PE intake (g/kg/day)	0.77 ± 0.32	0.86 ± 0.28	0.68 ± 0.34	0.094
GMPs intake (g/day) *	12.4 ± 2.2	12.1 ± 2.9	13 ± 1.4	0.711
GMPs (g/kg/day)/ total PE (g/kg/day) (%) *	57 ± 32.3	54 ± 13.3	61 ± 34.9	0.473
Total caloric intake (Kcal/Kg/day)	47.1 ± 17.1	58.7 ± 17.2	36.8 ± 8.0	**<0.001**
Phe intake (mg/kg/day)	13.4 [7.6–20.6]	16.9 [10.76–21]	9 [5.4–17.7]	**0.034**
Phe levels	389.7 ± 199	313.5 ± 106.8	457.9 ± 237.5	**0.013**
Patients non-compliant with diet (%)	9/36 (25%)	3/17 (18%)	6/19 (32%)	0.451

Data expressed as mean ± standard deviation or median [interquartile range]. * 5 observations (3 p and 2 P).

**Table 2:** Parameter averages estimated in the pre/post-pubertal and compliant/non-compliant groups through multivariate analysis, adjusting for sex.

Parameter	p-Group Compliant (a)	p-Group Non-Compliant (b)	P-Group Compliant (c)	P-Group Non-Compliant (d)	(a) vs. (b)	(c) vs. (d)	(a) vs. (c)	(b) vs. (d)
	LS. Means ± Standard Error				Adjusted *p*-Value *			
**BMI**	17.4 ± 0.9	19.3 ± 1.9	21.3 ± 0.8	25.2 ± 1.2	0.818	0.067	**0.018**	0.059
**sds-BMI**	−0.1 ± 0.3	0.7 ± 0.7	0.2 ± 0.3	0.8 ± 0.4	0.761	0.652	0.911	0.997
**FM** **n.v. 5.51–9.51** ***n.v. 10.01–20.07***	7.5 ± 2.4	12.9 ± 4.6	12.2 ± 2.2	11.9 ± 3.4	0.765	1.000	0.492	0.998
**PBF%** **n.v. 11.75–21.75** ***n.v. 13.89–23.89***	27.2 ± 4.4	28.9 ± 8.5	21.8 ± 4	13.5 ± 6.2	0.998	0.687	0.798	0.463
**TBW** **n.v.14.41–17.63** ***n.v. 35.38–43.26***	15 ± 2	18.3 ± 3.7	30.9 ± 1.8	45.6 ± 2.7	0.881	**0.001**	**<0.001**	**<0.001**
**WHR** **n.v.0.76–0.86** ***n.v. 0.78–0.88***	0.74 ± 0.02	0.8 ± 0.05	0.85 ± 0.02	0.83 ± 0.03	0.710	0.930	**0.010**	0.957
**MM** **n.v. 9.71–11.87** ***n.v. 26.77–32.78***	10 ± 1.6	12.8 ± 3.1	23 ± 1.4	35.3 ± 2.3	0.873	**0.001**	**<0.001**	**<0.001**
**sds-BQI**	−1.6 ± 0.4	−1.9 ± 0.9	−1.8 ± 0.3	−0.9 ± 0.5	0.984	0.496	0.978	0.755
**BQI**	54.8 ± 4.3	50.1 ± 10.6	69.6 ± 4	75.4 ± 5.9	0.978	0.850	0.081	0.182
**Leucine**	135 ± 11	106 ± 27	141 ± 10	147 ± 15	0.770	0.991	0.980	0.573
**Isoleucine**	69.9 ± 4.7	54.6 ± 12	76.6 ± 4.7	65.9 ± 6.7	0.667	0.570	0.755	0.847
**Valine**	227 ± 12	205 ± 30	243 ± 12	231 ± 17	0.908	0.940	0.795	0.874

* Adjusted for multiple comparison by Tukey. Block letters: range of normal values for prepubertal children. Italics: range of normal values for post-pubertal children.

**Table 3:** Parameters of the five patients analyzed before and after puberty.

General Characteristics												BIA Parameters										QUS Parameters			
Pts	Sex	Age		Tunner Stage		Phe Values (Mean SD)		BMI		SDS BMI		FM		PBF		TBW		WHR		MM		BQI		SDS-BQI	
1	F	9.3	11.5	I	III	217	148	22.15	26.21	1.31	1.62	20.70	21.80	53.70%	40.60%	13.20	23.50	0.75	0.87	8.60	16.80	54.26	75	0.90	−0.30
2	F	10.7	12.9	I	III	312	368	15.67	19.34	−1.22	−0.31	4.8	9.2	15.30%	20.9%	19.7	25.7	0.79	0.83	13.9	18.7	61.39	66.49	−1.70	−2.90
3	M	9.1	12.8	I	III	364	708	21.40	27.60	1.23	1.74	21.90	29.10	42.30%	42.80%	21.90	28.50	0.86	0.98	15.70	21.10	61.97	59.15	−1.00	−2.20
4	F	10.7	12.9	I	II	355	645	21.23	22.60	0.76	0.68	15.10	15.60	35.80%	29.8%	19.9	26.9	0.79	0.82	14	19.60	71.40	81	−1.40	−1.30
5	M	12.1	14.8	I	II	350	487	18.43	23.4	−0.39	0.66	13.5	19	39.10%	36.50%	15.5	24.3	0.76	0.85	10.6	17.5	52	65	−2.4	−2.3

Grey columns: prepubertal period. White columns: post-pubertal period.

## Data Availability

The data presented in this study are available upon request from the corresponding author.

## Supplementary Materials

The following supporting information can be downloaded at: https://www.mdpi.com/article/10.3390/children9091353/s1, Table S1: Comparison of BIA and QUS parameters according with gender.
