# Supplementary material for: Body Composition in Adolescent PKU Patients: Beyond Fat Mass
Source: Children (Basel). 2022 Sep 4;9(9):1353. doi: 10.3390/children9091353 (PMC9497631; doi:10.3390/children9091353)
Supplement: Supplementary file 1 [file children-09-01353-s001.zip › children-1851728-supplementary.pdf]

Table S1: Comparison of BIA and QUS parameters according with gender

	Males (n=16)	Females (n=20)	p
BMI	19,9±3,29	20,78±4,52	0,520
sds_BMI	0,93±1,34	1,31±1,3	0,391
FM	9,49±7,03	11,69±7,68	0,412
PBF_%	19,59±12,43	26,58±15,27	0,175
TBW	28,81±11,84	23,93±12,82	0,278
LBM	21,34±9,68	17,44±10,59	0,293
WHR	0,81 [0,77-0,86]	0,79 [0,76-0,84]	0,371
BQI	66,61±15,36	61,33±17,25	0,360
BQI SDS	-1,62 [-1,93;-1,17]	-1,6 [-2,27;-1,3]	0,890

Data expressed as mean±standard deviation or Median [Interquartile Range]
